# Galectin-3 Is a Natural Binding Ligand of MCAM (CD146, MUC18) in Melanoma Cells and Their Interaction Promotes Melanoma Progression

**DOI:** 10.3390/biom12101451

**Published:** 2022-10-10

**Authors:** Yaoyu Pang, Ellen Maxwell, Paulina Sindrewicz-Goral, Andrew Shapanis, Shun Li, Mark Morgan, Lu-Gang Yu

**Affiliations:** 1 Department of Biochemistry and Systems Biology, Institute of Systems, Molecular and Integrative Biology, University of Liverpool, Liverpool L69 3GE, UK; 2 Department of Physiology and Cell Signalling, Institute of Systems, Molecular and Integrative Biology, University of Liverpool, Liverpool L69 3GE, UK

**Keywords:** galectin-3, MCAM/CD146, melanoma, cancer, galectin–ligand interaction

## Abstract

Melanoma cell adhesion molecule (MCAM, CD146, MUC18) is a heavily glycosylated transmembrane protein and a marker of melanoma metastasis. It is expressed in advanced primary melanoma and metastasis but rarely in benign naevi or normal melanocytes. More and more evidence has shown that activation of the MCAM on cell surface plays a vital role in melanoma progression and metastasis. However, the natural MCAM binding ligand that initiates MCAM activation in melanoma so far remains elusive. This study revealed that galectin-3, a galactoside-binding protein that is commonly overexpressed in many cancers including melanoma, is naturally associated with MCAM on the surface of both skin and uveal melanoma cells. Binding of galectin-3 to MCAM, via *O*-linked glycans on the MCAM, induces MCAM dimerization and clustering on cell surface and subsequent activation of downstream AKT signalling. This leads to the increases of a number of important steps in melanoma progression of cell proliferation, adhesion, migration, and invasion. Thus, galectin-3 is a natural binding ligand of MCAM in melanoma, and their interaction activates MCAM and promotes MCAM-mediated melanoma progression. Targeting the galectin-3–MCAM interaction may potentially be a useful therapeutic strategy for melanoma treatment.

## 1. Introduction

Melanoma is one of the most common cancers in the world, particularly in Western countries [1]. Although the overall mortality rate of melanoma is relatively low in comparison with other common cancers, it still incurs substantial deaths each year. The melanoma cell adhesion molecule (MCAM, CD146, MUC18), a member of the immunoglobulin superfamily of cell adhesion molecules [2], is a marker of melanoma metastasis [3,4]. It is expressed in advanced primary melanoma and metastasis but rarely in benign naevi or normal melanocytes [3]. The expression of MCAM is associated with poor treatment outcome of melanoma patients [5].

Over the past years, increasing evidence has demonstrated that MCAM expression plays a vital role in melanoma progression and metastasis [4,6,7]. Vaccination against the MCAM showed to effectively protect mice from lethal challenges with melanoma in both primary and metastatic tumour models [8]. Intraperitoneal application of anti-MCAM antibody significantly inhibited melanoma growth and metastasis [9,10], whereas the depletion of MCAM completely abolished melanoma metastasis in mice [11]. MCAM-mediated melanoma progression is believed to be initiated through MCAM dimerization on cell surface [12,13], with subsequent induction of downstream AKT signalling, a feature that occurs at various stages of melanoma progression [14]. However, one key missing piece of information in MCAM-mediated actions in melanoma progression is the identity of the natural MCAM binding ligand that initiates MCAM activation.

Recently, while investigating the influence of galectin-3 on endothelial cell behaviours, we have revealed that galectin-3 can bind to the MCAM expressed on the surface of endothelial cells [15]. This induces MCAM dimerization and activation of AKT signalling in endothelial cells, leading to the increased secretion of several metastasis-promoting cytokines by endothelial cells [16]. Galectin-3 is a galactoside-binding protein and is commonly overexpressed by many types of cancers [17] including melanoma [18,19,20,21]. Overexpression of galectin-3 promotes a number of key steps in cancer progression and metastasis, e.g., adhesion, invasion, migration, and angiogenesis, through its interaction with various galactose-terminated cell surface glycans [17,22,23]. Suppression of the cellular expression of poly-N-acetyl-lactosamine, a disaccharide that is recognized by galectin-3 and occurs in various *N*-linked carbohydrate structures of cell membrane glycoproteins including MCAM, showed to substantially reduce melanoma metastasis in mice [24]. Pre-incubation of melanoma cells with a truncated form of galectin-3 or by feeding the mice with a galectin-3 inhibitor (modified citrus pectin) in drinking water was shown to also reduce melanoma metastasis [24].

These discoveries led us to speculate that galectin-3 may be the unidentified natural binding ligand of MCAM in MCAM activation and MCAM-mediated melanoma progression. A series of experiments were conducted in this study to test this possibility, and the results strongly support this notion.

## 2. Materials and Methods

### 2.1. Materials

Monoclonal antibodies against CD146/MCAM (mab 932) and galectin-3 (mab 1154), biotinylated goat anti-galectin-3 polyclonal antibody (BAF1154) were from R&D Systems (Abingdon, UK). Antibodies against AKT (9272S) and Phospho-AKT (Phospho T308) were purchased from Cell Signalling (Hitchin, UK). Antibody against CD146/MCAM rabbit polyclonal antibody was obtained from Proteintech (Rosemont, IL, USA). Protein A/G PLUS-Agarose beads were from Santa Cruz Biotechnology (Heidelberg, Germany). Calcein AM cell labelling solution was obtained from Invitrogen. *O*-glycanase (Endo-*O*-Glycosidase, GK80090) was purchased from Agilent (Santa Clara, CA, USA). ShRNA plasmid DNA for CD146/MCAM (TRCN0000151337), control shRNA plasmid (SHC002v), and *N*-glycanase PNGaseF (F8435) were from Sigma Aldrich (Dorset, UK). Matrigel matrix was obtained from Corning Life Sciences (Deeside, UK).

### 2.2. Cells

Human skin melanoma A375 cells (kindly provided by Dr. John Hikens, Netherland Cancer Institute, The Netherlands) were cultured in DMEM containing 200 mM L-glutamine, 0.4% penicillin and streptomycin, and 10% foetal calf serum (FCS). Human uveal melanoma Mel270 and 92.1 cells (kindly provided by Professor Sarah Coupland and Dr. Helen Kalirai, University of Liverpool, UK) were cultured in RPMI containing 200 mM L-glutamine, 0.4% penicillin and streptomycin, and 10% foetal calf serum.

### 2.3. Production of Recombinant Galectin-3 and Truncated Galectin-3

The full-length human galectin-3 and a C-terminal carbohydrate recognition domain of galectin-3 (Gal-3C, residues 115–250) were produced in *E. coli* as previously described [25,26].

### 2.4. Cell Proliferation

Sub-confluent cells were seeded into 96-well plates (5 × 10^4^ cells/well) and cultured at 37 °C. At a different time, 5 µL/well Calcein AM was added for 30 min before the cells were washed twice with PBS, and the fluorescence intensity was measured by a microplate reader (Tecan i-200, Tecan, Männedorf, Switzerland).

In some experiments, the cells were seeded and cultured for 2 days, and the medium was replaced with serum-free DMEM containing 0.25 mg/mL BSA for 16 h. Recombinant galectin-3 (5 or 10 µg/mL) or BSA (5 or 10 µg/mL, control) was then added, with or without 100 µg/mL lactose, for up to 72 h before the cells were washed and fluorescence intensity was measured.

### 2.5. Cell Migration

Cells (2 × 10^5^ cells/mL) were cultured in 3-well silicone inserts (ibidi GmbH, Gräfelfing, Germany) until monolayer formation. The medium was then replaced with a serum-free medium containing 0.25 mg/mL BSA for 16 h before the inserts were removed and 10 µg/mL galectin-3 or 10 µg/mL BSA was introduced. The gaps were imaged and measured every 12 h until closure.

### 2.6. Cell Adhesion

Twenty-four-well plates were coated with 250 µL pre-cooled Matrigel matrix (20 µg/mL) for 1 h at 37 °C. The plates were washed three times with PBS before introduction of 250 µL cell suspension (1 × 10^5^ cells/mL) with 10 µg/mL galectin-3 or 10 µg/mL BSA. After incubation for 30 min at 37 °C, the plates were gently washed three times with PBS, and the adhesion cells between three and five randomly selected fields/well were counted under a microscope (Olympus B51, Tokyo, Japan) with a 20× objective.

### 2.7. Cell Invasion

Transwell inserts in 24-well plates were incubated with a 100 µL/insert Matrigel matrix (20 µg/mL) for 2 h at 37 °C. After gentle wash with PBS, 150 µL/well cell suspension (1 × 10^5^ cells/mL) with 10 µg/mL galectin-3 or 10 µg/mL BSA in DMEM containing 1% FCS was added to the transwells, and a 500 µL culture medium containing 10% FCS was added to the bottom wells of the plates. After 16 h incubation at 37 °C, the Matrigel and uninvaded cells in the inserts were gently removed with cotton swaps. The inserts were washed once with PBS and fixed in 2% formaldehyde/PBS for 20 min. After washing with PBS, the inserts were stained with 5% crystal violet dye for 5–10 min. The insert membrane was removed by a scalpel and mounted to slides, and the stained cells at the bottom side of the membrane between three and five randomly selected fields/well were counted under a microscope (Olympus B51, Olympus, Hamburg, Germany) with a 20× objective.

### 2.8. Colony Formation

Sub-confluent cells were seeded (100 cells/well) into a 6-well plate and cultured for two days at 37 °C. Recombinant galectin-3 (10 µg/mL) or BSA (10 µg/mL) was then added to the culture medium every 48 h. After 10 days of culture, the cells were fixed with 2% paraformaldehyde/PBS for 15 min before being stained with 0.5% crystal violet for 1 h. After two washes with PBS, cell colonies containing >50 cells were counted.

### 2.9. ShRNA MCAM Suppression

MCAM shRNA or control shRNA plasmids (100 ng) were mixed with a Metafectin transfection reagent (1:4) in a serum-free, antibiotic-free DMEM for 30 min before introduction to 50% confluent A375 cells in an antibiotic-free, serum-containing DMEM in a 96-well plate. After 6 h culture at 37 °C, the media were replaced with a fresh DMEM containing 10% serum and 0.5 µg/mL puromycin for 72 h. The surviving cells were released from the plates by trypsinization, suspended in very low density, and seeded into 96-well plates. Wells containing a single cell were identified under a microscope and allowed to proliferate before they were analysed for MCAM expression by immunoblotting. The two selected subclones of MCAM-suppressed cells (55.6 and 55.3) were maintained in the same culture condition as the parental A375 cells.

### 2.10. MCAM Dimerization

A375 cells were cultured in 12-well plates (5 × 10^4^ cells/well) for 24 h before introduction of galectin-3 (10 µg/mL) or BSA (10 µg/mL) for 30 min at 37 °C. The cells were washed in ice-cold Ca2+- and Mg2+-free PBS and incubated with 3 mM BS3 cross-linker on ice for 30 min. After excess BS3 was quenched with 250 mM glycine for 10 min at 4 °C, the cells were lysed in an SDS-sample buffer without β-mercaptoethanol and analysed by immunoblotting.

### 2.11. Immunofluorescence and Confocal Microscopy

Sub-confluent cells cultured on glass coverslips in 24-well plates were washed with PBS and fixed with 4% paraformaldehyde for 15 min. After two washes with PBS, the cells were incubated with a blocking buffer (2% BSA in PBS) before incubation with mouse anti-galectin-3 monoclonal antibody and rabbit anti-MCAM polyclonal antibody (Proteintech) (1:500 and 1:1000 dilution, respectively, in 1% BSA in PBS) for 2 h at room temperature. Control wells were incubated with 1% BSA in PBS. After three washes with PBS, the cells were incubated with Texas Red-conjugated anti-mouse antibody (Vector Laboratories, Burlingame, CA, USA) at 1:300 dilution and FITC-conjugated anti-rabbit (Dako Agilent, Santa Clara, CA, USA) at 1:300 dilution for 1 h at room temperature. The cells were washed five times with PBS before being mounted with DAPI-containing fluorescent mounting media (Vector Laboratories, Burlingame, CA, USA) and analysed by a Zeiss LSM 800 Airyscan confocal microscope.

In some experiments, the cells were treated with 10 µg/mL recombinant galectin-3 or 10 µg/mL BSA for 1 h before they were fixed and analysed for MCAM and galectin-3 localization by confocal microscopy.

### 2.12. Co-Immunoprecipitation

Sub-confluent cells (~4 × 10^6^) in T-75 flasks were washed twice with ice-cold PBS before being lysed with 1 ml of a RIPA buffer (150 mM NaCl, 50 mM Tris–HCl, 1% NP40, 1% protease inhibitor cocktail, pH 7.4) for 10 min on ice. After centrifugation of the lysate at 100,000× *g* for 10 min at 4 °C, the supernatant was mixed with 20 µL protein A/G PLUS-agarose beads for 1 h on a rotary shaker at 4 °C. The beads were removed by centrifugation at 1000× *g* for 3 min at 4 °C. The cleaned supernatant was collected, and 0.5 mL supernatant was incubated with 5 µg anti-MCAM antibody or 5 µg mouse immunoglobulin on a rotary shaker for 1 h at 4 °C before introduction of Protein A/G agarose beads (20 µL) overnight at 4 °C. After centrifugation at 1000× *g* and removal of the supernatant, the pellets were washed twice with an ice-cold RIPA buffer and twice with ice-cold PBS before the addition of 50 µL PBS and 50 µL SDS-sample buffer and subsequent analysis by immunoblotting.

### 2.13. Protein De-Glycosylation

Sub-confluent A375 cells were washed twice with PBS and incubated with galectin-3 (5 µg/mL) in a serum-free medium for 2 h at 37 °C. The cells were washed with PBS and then lysed with a lysis buffer (1% Triton X-100 in PBS) for 15 min on ice. After centrifugation at 4500× *g* rpm for 10 min, the supernatant was collected and aliquoted to four 1 mL aliquots. Three aliquots were incubated with mouse monoclonal anti-MCAM antibody (R&D Systems) at 2 µg/mL and one incubated with control mouse immunoglobulin (2 µg/mL) overnight at 4 °C. Eighty microlitres of protein A/G Plus-agarose beads was then added to each aliquot, incubated on a roller for 4 h at room temperature. After two washes with PBS, one MCAM antibody-treated aliquot was incubated with *N*-glycanase PNGaseF (5U) and one with *O*-glycanase (10 mU) overnight at 37 °C. The third aliquot and the immunoglobulin control aliquot were incubated with PBS. After three washes with PBS, the proteins were released from the beads using an SDS-sample buffer and analysed by immunoblotting. Galectin-3 immunoblotting was carried out using biotinylated, goat anti-galectin-3 polyclonal antibody at 1:4000 dilution, followed by Avidin-Peroxidase at 1:8000. MCAM immunoblotting was performed using mouse anti-MCAM monoclonal antibody at 1:5000 dilution and followed by peroxidase-conjugated anti-mouse antibody (Santa Cruz, CA, USA) at 1:8000. The blots were developed using a chemiluminescence Super Signal kit and visualized with the Molecular Imager^®^ Gel Doc™ XR System (Biorad, Hercules, CA, USA).

### 2.14. Statistical Analysis

One-way analysis of variance (ANOVA) followed by Bonferroni correction was used for multiple comparisons. Differences were considered significant when *p* < 0.05.

## 3. Results

### 3.1. Galectin-3 Is a Natural Ligand of MCAM in Human Melanoma Cells

**Figure 1:**
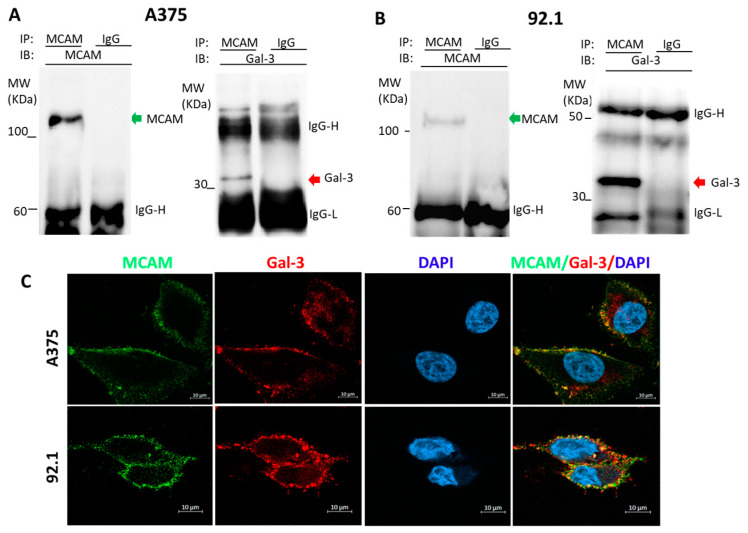
Galectin-3 is a natural ligand of MCAM in human melanoma cells. (**A**,**B**): MCAM and galectin-3 are naturally associated in human skin A375 (**A**) and Uveal 92.1 melanoma (**B**) cells. A375 and 92.1 cells were lysed and immunoprecipitated with antibodies against MCAM or galectin-3 and followed by immunoblotting. (**C**) MCAM and galectin-3 are co-localized on surface of melanoma cells. Localization of MCAM (green) and galectin-3 (red) in A375 and 92.1 cells was analysed by confocal microscopy following immunofluorescence staining (cell nucleus were stained by DAPI as blue). Representative images from at least two independent experiments are shown.

To determine the possible association of galectin-3 and MCAM in melanoma, two types of human melanoma cells, skin A375 and uveal 92.1 cells, were selected and investigated in this study. MCAM immunoprecipitation revealed the presence of galectin-3 in the MCAM immunoprecipitates of both A375 (Figure 1A) and 92.1 (Figure 1B) cells. Immunofluorescence staining of MCAM and galectin-3 followed by confocal microscopy showed the co-localization of galectin-3 and MCAM on the cell surface of both A375 and 92.1 cells (Figure 1C). These indicate that MCAM and galectin-3 are naturally associated on the cell surface of melanoma cells.

### 3.2. Galectin-3 Binding to MCAM Induces MCAM Dimerization and Subsequent Downstream AKT Signalling

**Figure 2:**
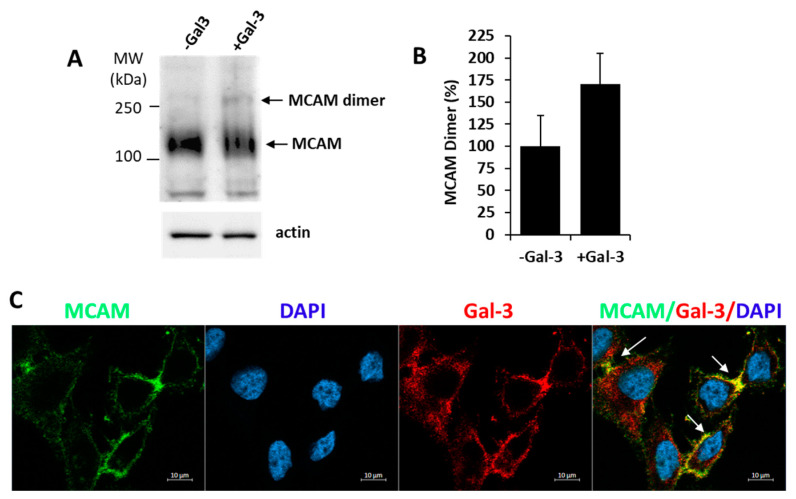
Galectin-3 induces MCAM dimerization and clustering on melanoma cell surface. (**A**) Galectin-3 induces MCAM dimerization in A375 cells. Cells were treated with 10 µg/mL galectin-3 or BSA (control) for 30 min before the cells were treated with BS3 cross-linker and analysed by MCAM immunoblotting. Representative blots are shown in (**A**) and percentage change of MCAM dimer bands in response to galectin-3 from two independent experiments is shown in (**B**). (**C**) Galectin-3 induces MCAM clustering on surface in A375 cells. Cells were treated with 10 µg/mL galectin-3 for 1 h, fixed and immunofluorescence-stained for MCAM (green) and galectin-3 (red), and imaged by confocal microscopy (cell nuclei were stained by DAPI as blue) (arrows point to clustering MCAM).

As MCAM dimerization is known to occur in the activation of MCAM in melanoma [3], we then investigate the influence of exogenous introduction of galectin-3 on MCAM dimerization. It was found that the introduction of exogenous galectin-3 caused a 70% increase of MCAM dimer formation in A375 cells (Figure 2A,B). Moreover, confocal microscopy analysis showed clustering of MCAM with galectin-3 on the cell surface in cell response to the introduction of galectin-3 (Figure 2C). These results suggest that binding of galectin-3 to MCAM promotes MCAM dimerization and clustering on the cell surface.

**Figure 3:**
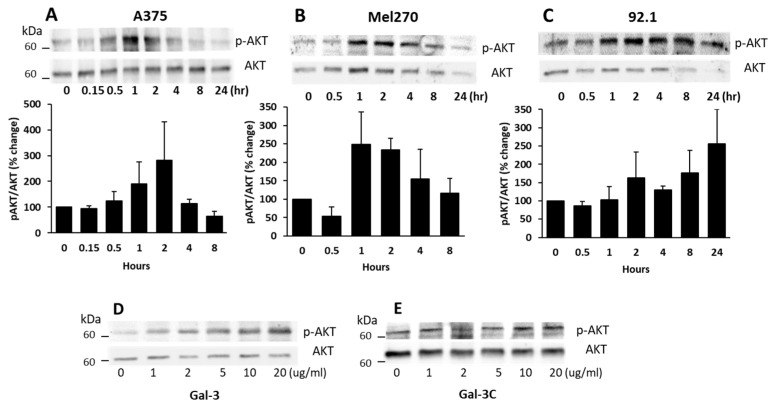
Galectin-3–MCAM interaction induces AKT activation in melanoma cells. (**A**–**C**) Galectin-3 induces time-dependent increase of AKT phosphorylation in A375 (**A**), Mel270 (**B**), and 92.1 (**C**) cells. Cells were treated with 10 µg/mL galectin-3 for different times before the cells were lysed, and p-AKT and AKT levels were analysed by immunoblotting. Band densities from two independent experiments were quantified and expressed as percentage p-AKT/AKT changes. (**D**,**E**) Full-length galectin-3, but not the truncated, C-terminal galectin-3 (Gal-3C), induces AKT activation in A375 cells in dose-dependent manner. Cells were treated with different concentrations of either full-length Gal-3 (**D**) or Gal-3C (**E**) for 1 h before cells were lysed and p-AKT and AKT levels were determined by immunoblotting.

Many previous studies have shown that MCAM activation in melanoma cells is closely associated with the induction of AKT signalling [14]. To see whether the galectin-3–MCAM interaction on cell surface affects downstream MCAM signalling, AKT activation was assessed in cell response to exogenous galectin-3. It was found that the introduction of galectin-3 to A375 (Figure 3A), Mel270 (Figure 3B), and 92.1 (Figure 3C) cells caused a time-dependent increase in AKT phosphorylation of all three melanoma cells with peak AKT activation at 1–2 h. This effect of galectin-3 on AKT phosphorylation was seen to also occur dose-dependently (Figure 3D) at galectin-3 concentrations similar to that seen in the circulation of cancer patients with metastasis [27]. The effect of galectin-3 on AKT activation occurred only with full-length galectin-3 (Figure 3D) but not with the N-terminal-truncated, C-terminal carbohydrate recognition domain of galectin-3 (Gal-3C) (Figure 3E). As the N-terminal domain of galectin-3 controls galectin-3-mediated receptor clustering [17], the lack of effect of Gal-3C on AKT phosphorylation is in keeping with the importance of galectin-3-mediated MCAM clustering in MCAM activation and downstream signalling. Together, these results indicate that galectin-3 binding to the MCAM, which induces MCAM dimerization and clustering on the cell surface, activates downstream AKT signalling in melanoma cells.

### 3.3. Galectin-3 Binds to MCAM via O-Linked Glycans

**Figure 4:**
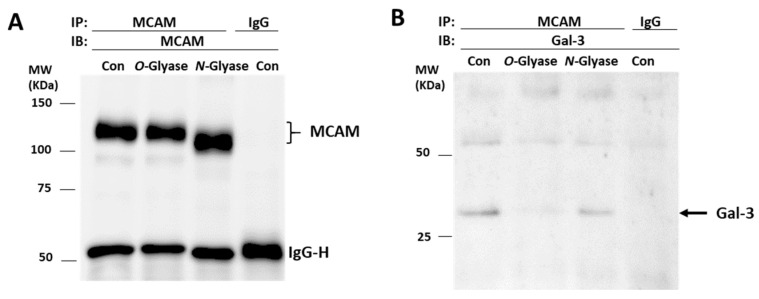
Galectin-3 binds to MCAM via *O*-glycans. A375 cells were treated without or with *N*- or *O*-glycanase before cells were lysed and immunoprecipitated with MCAM antibody or control immunoglobulin. Immunoprecipitates were analysed by immunoblotting with antibodies against MCAM (**A**) and galectin-3 (**B**). Treatment of the cells with *O*-, but not *N*-, glycanase reduces presence of galectin-3 in MCAM immunoprecipitates.

To gain insight into the molecular interaction between galectin-3 and MCAM, A375 cells were treated with *N*-glycanase PNGaseF (which can remove almost all *N*-linked glycans) or *O*-glycanase (which cleaves unsubstituted Galβ1,3GalNAc-α-O-) and galectin-3–MCAM interaction then analysed by MCAM immunoprecipitation and immunoblotting. Treatment of the cells with *N*-glycanase, but not *O*-glycanase, led to MCAM shift in electrophoresis (Figure 4A), similarly as the behaviour of the endothelial associated MCAM in response to treatment with *N*- and *O*-glycanases [15]. The presence of galectin-3 in MCAM immunoprecipitates was shown to be almost completely depleted in *O*-glycanase-treated cells in comparison with that in the control cells (Figure 4B). Treatment with *N*-glycanase appeared to also result in a small reduction of galectin-3 presence in the MCAM immunoprecipitates in comparison with the control cells. These indicate that galectin-3 interaction with the MCAM in melanoma cells is predominately via galectin-3 binding to *O*-linked glycans with perhaps also some contribution of the *N*-linked glycans on the MCAM. The observed molecular shift of the MCAM in response to treatment with *N*-glycanase but not *O*-glycanase also suggests that the MCAM in melanoma cells likely carries more *N*-linked than unsubstituted Galβ1,3GalNAcα-O-carbohydrate structures.

### 3.4. Suppression of MCAM Expression by shRNA Abolishes Galectin-3-Induced AKT Activation

**Figure 5:**
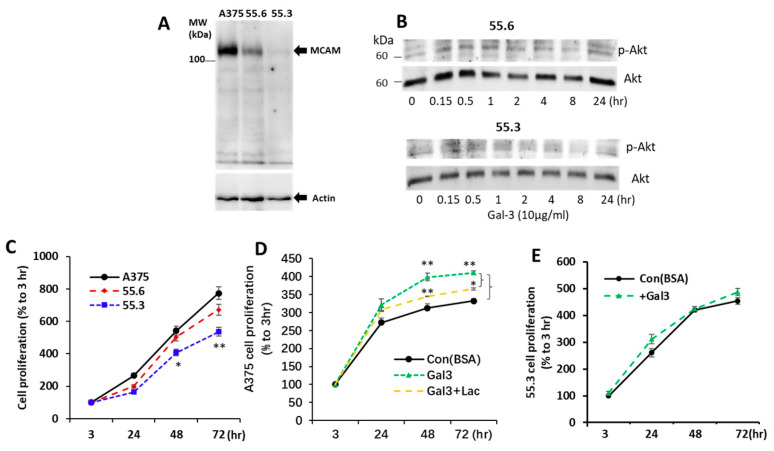
MCAM shRNA suppression inhibits AKT activation and proliferation of melanoma cells in response to galectin-3. (**A**) Generation of two subclones (55.6 and 55.3) of MCAM suppressed A375 cells with shRNA (**A**). (**B**) MCAM suppression reduces AKT activation in A375 cell in response to galectin-3. Moderately MCAM-suppressed (55.6) and highly MCAM-supressed (55.3) subclones of A375 cells were treated with 10 µg/mL galectin-3 for different time before the cells were lysed and levels of p-AKT and AKT were determined by immunoblotting. Representative blots from two independent analyses are shown. (**C**–**E**) Galectin-3–MCAM interaction increases melanoma cell proliferation. Proliferation of parental or MCAM suppressed A375 cells was analysed in the absence (**C**) or presence of Gal-3 (**D**,**E**) or lactose (**D**). Data are presented as mean ± SEM from three independent experiments, each in triplicate. * *p* < 0.05, ** *p* < 0.01.

To confirm the effect of galectin-3 on AKT phosphorylation was the consequence of galectin-3 interaction with the MCAM, MCAM expression in A375 cells was suppressed by shRNA, and two A375 subclones of 55.6 and 55.3 cells were generated. MCAM expression in 55.6 and 55.3 cells was reduced by 68% and 91%, respectively, in comparison with parental A375 cells (Figure 5A). Although galectin-3 induced a time-dependent increase in AKT phosphorylation in parental A375 cells (Figure 3A), this effect of galectin-3 was reduced in MCAM moderately suppressed 55.6 cells and completely disappeared in MCAM highly suppressed 55.3 cells (Figure 5B). This provides further support to the interaction of galectin-3 with the MCAM on the activation of downstream MCAM signalling in melanoma cells.

### 3.5. Galectin-3–MCAM Interaction Promotes Melanoma Cell Proliferation, Adhesion, Migration, and Invasion

To investigate the influence of the galectin-3–MCAM interaction on melanoma cell behaviours, melanoma cell proliferation, migration, adhesion, and invasion of A375 and MCAM-knockdown 55.6 and 55.3 cells in response to galectin-3 were analysed. Suppression of MCAM expression was associated with significant reduction of melanoma cell proliferation (Figure 5C). Introduction of galectin-3 enhanced the proliferation of A375 cells, an effect that was inhibited by the presence of a galectin-3 inhibitor lactose (Figure 5D). Introduction of galetin-3, however, did not affect the proliferation of MCAM-suppressed 55.3 cells (Figure 5E).

**Figure 6:**
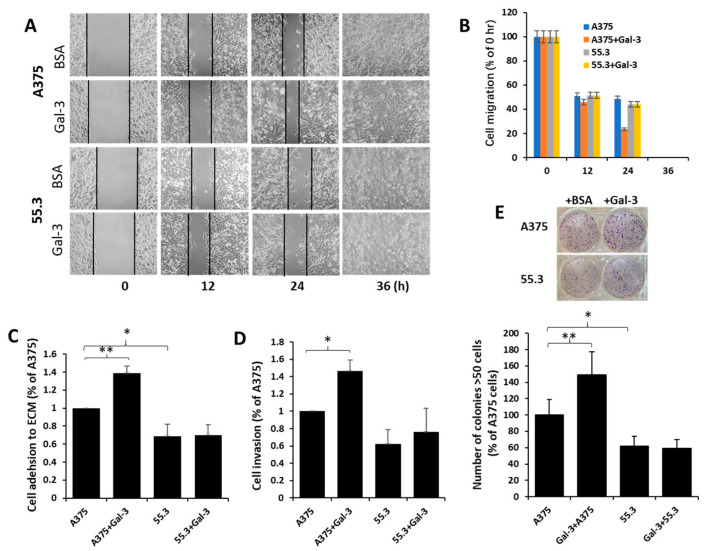
Galectin-3–MCAM interaction promotes melanoma cell adhesion, migration, invasion, and colony formation. (**A**) MCAM–galectin-3 interaction increases melanoma cell migration. Gaps of A375 and 55.3 cell monolayer were created by silicone inserts and imaged every 12 h in the presence of 10 µg/mL galectin-3 or BSA (control). Representative images are shown in (**A**), and percentage of the gaps in comparison with that at 0 h from two independent experiments are shown in (**B**). Gap of A375 cells treated with galectin-3 is 51% smaller than that of A375 cells without galectin after 24 h. (**C**,**D**) Galectin-3–MCAM interaction promotes melanoma cell adhesion to and invasion through Matrix proteins. A375 and 55.3 cell adhesion to or invasion through matrix proteins in the presence or absence of 10 µg/mL galectin-3 was assessed. (**E**) MCAM–galectin-3 interaction enhances melanoma colony formation. Number of colonies (>50 cells) after 10-day culture in the presence of 10 µg/mL galectin-3 or BSA were quantified. Data are presented as mean ± SEM of at least three independent experiments, each in triplicate. * *p* < 0.05, ** *p* < 0.01.

Suppression of MCAM was seen to also be associated with the reduction of cell migration (Figure 6A,B), cell adhesion to (Figure 6C), and invasion through (Figure 6D) matrix proteins in comparison with the parental A375 cells. Introduction of galectin-3 increased the migration, adhesion, and invasion of parent A375 cells but not MCAM-suppressed 55.3 cells (Figure 6A–D). Furthermore, a higher number of colonies were formed in parental A375 than in MCAM-suppressed 55.3 cells, and the introduction of galectin-3 increased the colony formation of A375 but not 55.3 cells (Figure 6E). Together, these results indicate that the galectin-3–MCAM interaction promotes melanoma cell proliferation, adhesion, migration, and invasion.

## 4. Discussion

This study showed that galectin-3 is naturally associated with MCAM on the surface of both skin and uveal melanoma cells. Binding of galectin-3 to the MCAM, via *O*-linked glycans on MCAM, induces MCAM dimerization and clustering on the cell surface and subsequent activation of downstream AKT signalling. This leads to increased melanoma cell proliferation, adhesion, migration, and invasion.

Despite more and more studies reporting the importance of MCAM expression and activation in melanoma progression and metastasis [3,4,6,7], the natural binding ligand that induces MCAM activation in melanoma so far remains largely elusive. The MCAM has been reported to be recognized by a few molecules in other cell types such as endothelial and immune cells. In endothelial cells, where the MCAM used to be considered as an endothelial marker for the identification of circulating endothelial cells [28], binding of VEGFR-2 to the MCAM was shown to activate AKT signalling and increase endothelial cell migration [29]. Binding of Netrin-1 to the MCAM in endothelial cells was reported to activate an array of downstream signalling pathways and increase endothelial cell proliferation, migration, and angiogenesis [30]. Interaction of the cell surface MCAM with exogenous galectin-1 in endothelial cells showed to protect cells against galectin-1-induced cell apoptosis [31]. Interaction of Laminin-411 with the MCAM on immune cells has been reported to facilitate Th17 cell entry into the central nervous system [32]. In this study, galectin-3 was shown to be naturally associated with MCAM in melanoma cells, and their interaction promotes melanoma cell proliferation, adhesion, migration, and invasion, all of which are important steps in melanoma progression. MCAM has been reported to be recognized by galectin-1 in melanoma cells, and their interaction increased melanoma cell migration [33]. As galectin family members share similar carbohydrate recognition domains and can often, although with different affinities, bind similar carbohydrate structures [34], it is very possible that the MCAM may be bound by several galectin members in melanoma, and their interactions may all influence melanoma development and progression.

This study showed that the galectin-3–MCAM interaction promotes MCAM dimerization and clustering on the cell surface, leading to the activation of AKT signalling. Although the activation of AKT signalling is believed to be critical in MCAM-mediated melanoma progression [14], several other singling pathways have been reported to also occur following MCAM activation. For example, the VEGF/FAK network was reported to be activated in MCAM signalling and promotes melanoma extravasation across vascular barriers in melanoma cell haematogenous metastasis to the lung [7]. MCAM-mediated activation of PI4K signalling was reported to help recruitment of actin-linking ezrin–radixin–moesin to cell protrusions and promote melanoma cell motility [35]. It is possible that galectin-3–MCAM interaction-mediated effects on melanoma cell proliferation, adhesion, and invasion may also involve the activation of other downstream signalling pathways in addition to AKT pathways.

Galectin-3 is a multifunctional protein and is involved in various steps in cancer development, progression, and metastasis by binding to a number of galactose-terminated, *N-* and *O*-linked cell surface glycans [17,36]. For example, galectin-3 can bind to the galactose-β1,3N-acetyl-galactosamineα-Thr/Ser, an oncofetal carbohydrate antigen (Thomsen-Friedenreich antigen, TF antigen) that occurs in >90 tumour cells [37,38] and can be proteolytically removed by *O*-glycanase [39]. MCAM is known to be heavily modified by glycosylation of both *N*- (eight sites) and *O*-linked glycans [13,33] with carbohydrates making upto 35% its molecular weight [40]. Our early study showed that in normal endothelial cells, binding of galectin-3 to the MCAM, which induces endothelial cell secretion of cytokines such as IL-6 and G-CSF [16], is mediated by galectin-3 binding to the *N*-glycans on MCAM [15]. In the present study, the interaction between galectin-3 and the MCAM in melanoma cells was seen to be mediated largely via *O*-linked carbohydrates on the MCAM. Such a difference of galectin-3 binding to the MCAM in melanoma and normal endothelial cells is very interesting. Given that changes of the glycosylation of cell membrane glycoproteins very commonly occur in cancer cells including melanoma [41], the binding difference of galectin-3 with the MCAM observed in melanoma and normal endothelial cells suggests possible difference of glycosylation structures of the MCAM in cancer and physiological conditions or between melanoma and endothelial cells. The revelation by an early NMR study showing the existence of two galectin-3 binding sites on MCAM [42], one involves the S- face and one F-face of the galectin-3 sugar-binding β-sheets, is in keeping with the multimode interactions of galectin-3 with the MCAM.

Receptor clustering is a common feature of galectins after their binding to cell surface receptors [43]. For example, binding of galectin-3 to the oncofetal galactose-β1,3N-acetyl-galactosamineα-Thr/Ser on the transmembrane mucin protein MUC1 in cancer cells induces MUC1 cell surface polarization. This exposes the cell surface adhesion molecules, resulting in the increase of tumour cell–cell aggregation and of tumour cell–endothelial cell interaction [44]. Galectin-3-mediated MUC1 clustering also increases the interaction of MUC1 with EGFR on the cell surface, resulting in an increased responsiveness of EGFR dimerization and activation in response to EGF binding [45]. It is therefore not surprising that binding of galectin-3 to the highly glycosylated MCAM in melanoma cells also causes MCAM clustering on the cell surface. An increased proximity of MCAMs in clusters on the cell surface in response to galectin-3 binding would likely help the formation of MCAM dimers and subsequent activation of downstream signalling. The binding affinity between galectin-3 and a recombinant form of the extracellular domain of the MCAM was reported to be at a sub-micromolar level [42]. It will be interesting in future research to determine whether the strength of galectin-3 interaction with the melanoma-derived MCAM is higher than its interaction with the recombinant form of MCAM using biophysical approaches such as isothermal titration calorimetry and surface plasmon resonance. Such investigations may help to gain insight into the role of MCAM glycosylation in MCAM-mediated actions in melanoma progression.

Overall, galectin-3 is identified in this study as a natural ligand of the MCAM in melanoma cells, and their interaction induces MCAM clustering and subsequent AKT activation. This leads to the increases in a number of steps in the melanoma progression of cell proliferation, adhesion, migration, and invasion. Targeting the galectin-3–MCAM interaction therefore may potentially be a useful therapeutic strategy for melanoma treatment.

## Data Availability

Not applicable.

## Abbreviations

BSA, bovine serum albumin; FCS, foetal calf serum; Gal-3, galectin-3; Gal-3C, C-terminal galectin-3; MCAM, melanoma cell adhesion molecule (CD146, MUC18).
